# Management Dilemma of an Asymptomatic Carotid Web in a High-Risk Patient: A Case Report

**DOI:** 10.7759/cureus.105420

**Published:** 2026-03-18

**Authors:** Elias M Nabhan, Edouard Naoum Nehme, Alexandrina-Paula Vana, Nabil Poulos

**Affiliations:** 1 Cardiology, Centre Hospitalier Josephine Baker, Gonesse, FRA

**Keywords:** carotid artery diaphragm, carotid bulb, carotid web, embolic stroke, internal carotid artery

## Abstract

Carotid webs, particularly in high-risk patients, can lead to ischemic cerebral events mainly through thrombus formation. While the management of symptomatic carotid webs is relatively well-established, the management of asymptomatic patients remains poorly represented in the vascular literature.

We report the case of a 44-year-old male smoker with a sedentary lifestyle and a strong family history of ischemic stroke who was referred by his general practitioner for routine vascular evaluation. Doppler ultrasound (DUS) of the supra-aortic vessels identified a shelf-like fibrous structure on the posterior wall of the proximal internal carotid artery (ICA) without stenosis or thrombus. Computed tomography angiography (CTA) confirmed the diagnosis of carotid web and excluded intraluminal thrombosis.

In the absence of neurological symptoms and given the strong family history of ischemic stroke, antiplatelet therapy with aspirin and intensive lifestyle modification were initiated. This case illustrates the diagnostic and therapeutic challenges associated with the incidental diagnosis of carotid webs in asymptomatic patients.

## Introduction

A carotid web is a focal intimal membranous projection extending from the posterior wall of the proximal internal carotid artery (ICA) into the arterial lumen. Histologically, it represents a localized form of intimal fibromuscular dysplasia composed primarily of fibrous tissue proliferation without lipid core or calcification typical of atherosclerosis [1,2].

Although first described in 1968, carotid webs gained a broader recognition in the early 2000s as an important cause of embolic ischemic stroke, particularly among younger patients with idiopathic anterior stroke [3,4]. Their prevalence in the general population remains unknown but is estimated to be less than 1%, with a higher prevalence reported among women of African descent [1]. Among patients younger than 65 years presenting with cryptogenic stroke, the reported prevalence is approximately 8-10% [5,6].

Unlike atherosclerotic plaques, carotid webs do not lead to a hemodynamically significant stenosis. Their clinical relevance appears related to local flow disturbance and relative blood stasis distal to the intimal projection, promoting thrombus formation and distal embolization [7].

Despite increasing recognition, optimal management remains unclear, particularly in asymptomatic patients. We present a case of an incidentally discovered carotid web in a young patient with a strong familial predisposition to stroke, highlighting the management dilemma of asymptomatic carotid webs.

## Case presentation

A 44-year-old male patient was referred by his general practitioner for a cardiovascular evaluation because of a significant family history of ischemic stroke. His father had a history of two ischemic strokes, and his elder brother died from stroke-related complications at the age of 52. The patient reported a 20 pack-year smoking history as well as a sedentary occupation in the information technology sector. He denied any history of prior transient ischemic attack, stroke, cervical trauma, connective tissue disease, or heavy weight lifting.

Physical examination was unremarkable, with normal blood pressure and cardiovascular findings. Laboratory results are summarized in Table 1.

**Table 1:** Laboratory findings. HDL: high-density lipoprotein, LDL: low-density lipoprotein

Parameter	Result	Reference Range
Hemoglobin	13 g/dL	12–16 g/dL
White blood cells	7 000 /µL	4000–10000 /µL
Creatinine	70 µmol/L	50–100 µmol/L
Total cholesterol	1.5 g/L	0-2 g/L
HDL cholesterol	0.80 g/L	≥ 0.4 g/L
LDL cholesterol	1.1 g/L	1-1.43 g/L
Triglycerides	1.02 g/L	0.61-1.49 g/L

**Figure 1:**
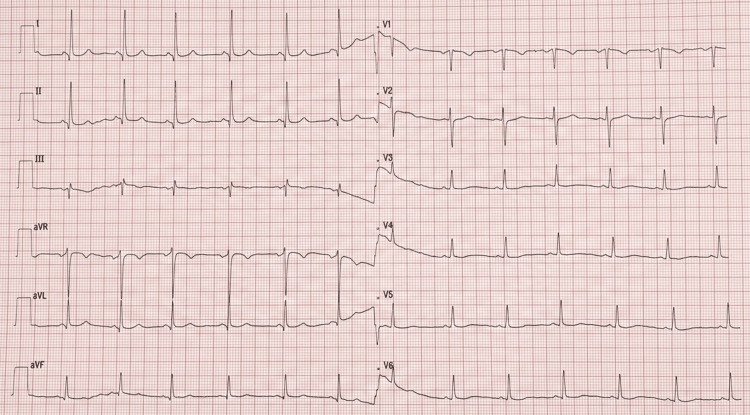
A 12-lead ECG demonstrating normal sinus rhythm. ECG: electrocardiogram

A 12-lead electrocardiogram (ECG) showed a normal sinus rhythm without conduction abnormalities (Figure 1).

**Figure 2:**
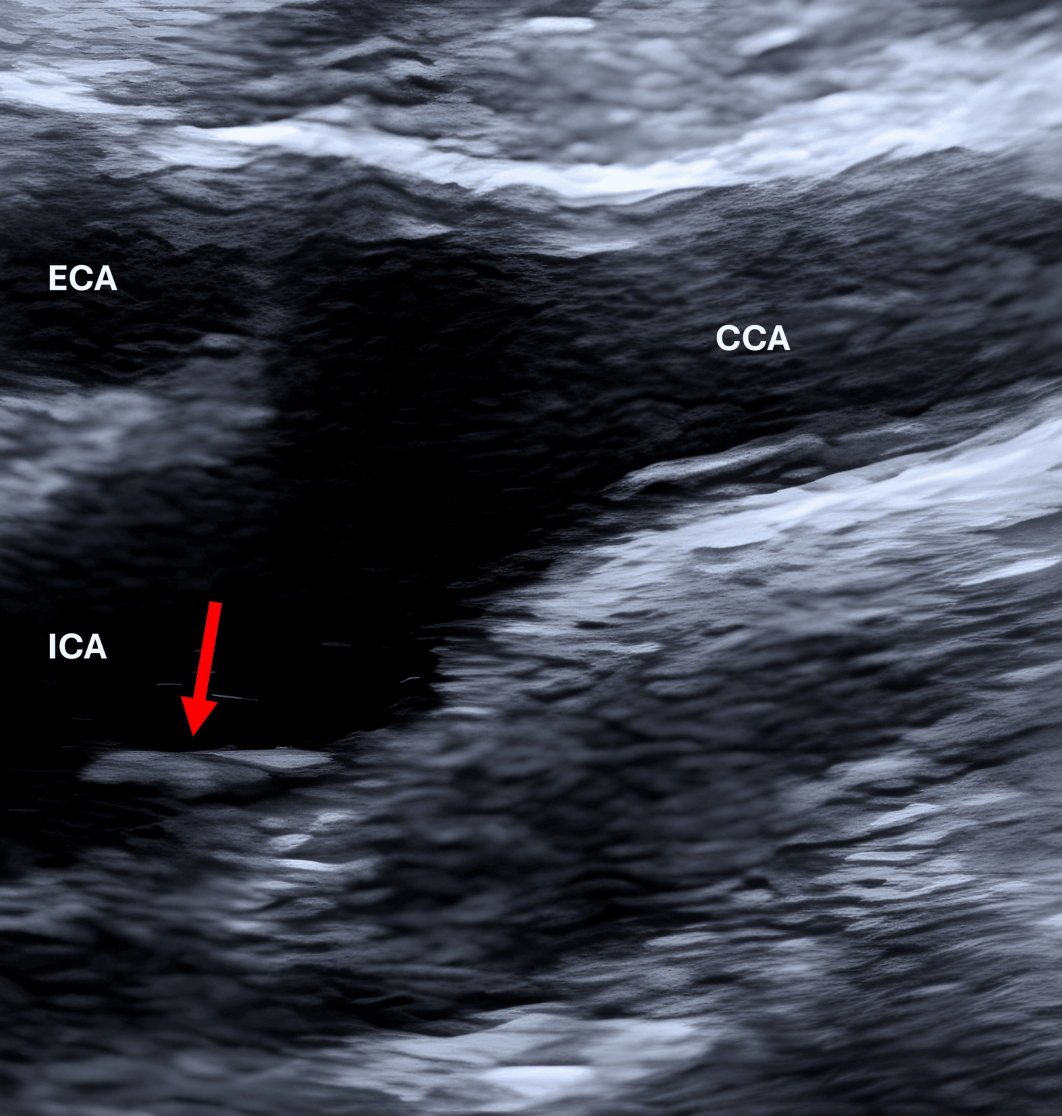
DUS showing shelf-like echogenic projection on the posterior wall of the left ICA (red arrow). DUS: Doppler ultrasound; ICA: internal carotid artery; ECA: external carotid artery; CCA: common carotid artery.

Doppler ultrasound (DUS) of the supra-aortic vessels revealed minimal intima-media thickening without significant stenosis or flow acceleration. A unilateral, shelf-like echogenic projection arising from the posterior wall of the proximal left ICA was identified, measuring approximately 3-4 mm in length and 2.5 mm in thickness. No thrombus or hemodynamic alteration was detected (Figure 2).

**Figure 3:**
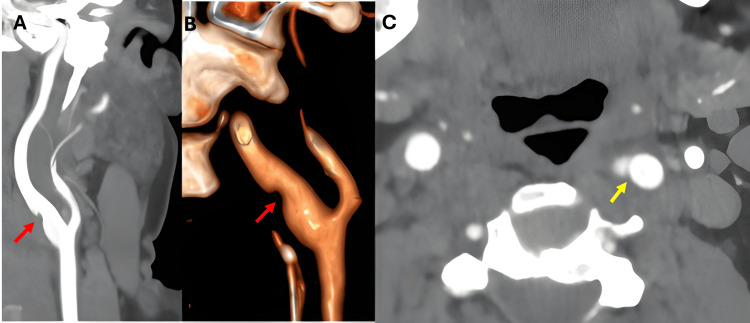
CTA of the neck showing the carotid web in the left ICA on coronal (A), 3D-reconstructed (B) views (red arrows), and axial view (C, yellow arrow). CTA: computed tomography angiography

Computed tomography angiography (CTA) of the neck confirmed a left-sided, unilateral, regular, non-calcified, membrane-like intraluminal projection originating from the posterior carotid bulb without significant luminal narrowing or thrombosis (Figure 3).

Given the strong family history of ischemic stroke, and despite the absence of neurological symptoms and the lack of thrombus, antiplatelet therapy with aspirin 75 mg once daily was initiated. Intensive lifestyle modification, including smoking cessation and structured physical activity, was strongly recommended. At the one-year follow-up, the patient remained asymptomatic with no neurological events. Repeat DUS demonstrated no progression of the lesion or thrombus formation.

## Discussion

**Figure 4:**
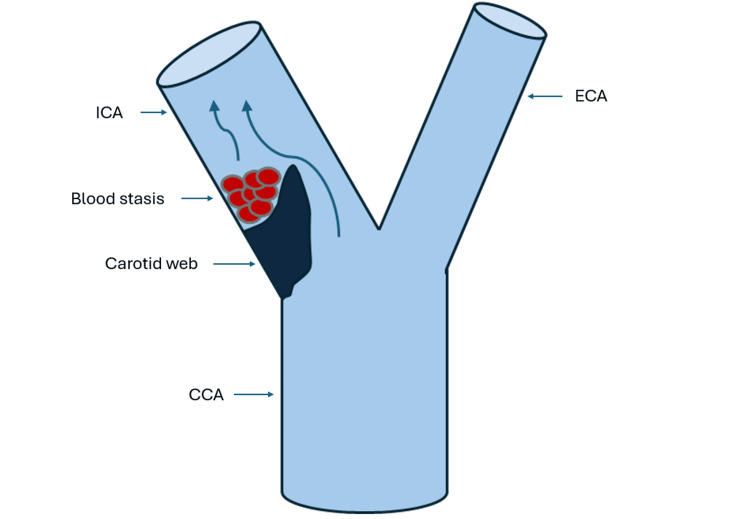
Schematic representation of hemodynamic disturbance and thrombus formation within the carotid web. ICA: internal carotid artery; ECA: external carotid artery; CCA: common carotid artery. This figure is an original illustration created by the authors using Microsoft PowerPoint (Microsoft Corporation, Redmond, WA, USA)

Carotid webs represent a rare form of intimal fibrous dysplasia that occur primarily in the carotid bulb [1,2]. In most cases, these lesions arise from the posterior carotid wall, which is subjected to complex hemodynamic forces. Computational flow analyses have demonstrated regions of flow separation and low wall shear stress distal to the web, creating a prothrombotic environment. In addition, the carotid web may act as a nidus for blood stasis and thrombus formation [7,8]. As a result, embolic events may occur despite minimal or absent stenosis. Figure 4 provides a schematic illustration of these mechanisms.

Several observational studies have shown an association between carotid web and embolic stroke. A systematic review reported high recurrence rates in symptomatic patients treated medically [5]. Cohort analyses have demonstrated increased risk of recurrent ischemic events despite antiplatelet therapy alone [9]. Carotid webs seem to be more frequent in younger patients with recurrent cryptogenic anterior stroke [6].

The differential diagnosis includes atherosclerotic plaque, carotid artery dissection, and intraluminal thrombus. It is important to accurately differentiate between the various types of pathology affecting the carotid artery. Most carotid webs originate from the posterior wall of the proximal internal carotid artery and, in general, they do not cause significant stenosis [1,2]. In contrast, atherosclerosis, carotid artery dissection, and intraluminal thrombus in the same location may result in significant stenosis or even complete occlusion [5,8].

On DUS, a carotid web appears as a smooth, shelf-like projection with no calcification [1,8]. However, atherosclerotic plaques are often irregular and frequently contain calcifications [10]. Dissection usually presents as an intimal flap, spiral configuration, and the presence of true and false lumens, whereas thrombi often appear irregular or mobile [8].

While atherosclerosis is a progressive disease, carotid webs are usually stable over time unless a superimposed thrombus develops [4]. In contrast, dissections and thrombi tend to evolve over time and may partially resolve [1,8].

Recognition of these features on DUS and CTA is critical. In our patient, the diagnosis was initially suspected on DUS through the identification of a shelf-like echogenic projection arising from the posterior wall of the proximal ICA and was subsequently confirmed on CTA, which demonstrated a smooth, non-calcified intraluminal membrane without associated thrombus or significant luminal narrowing.

In asymptomatic carotid webs with no thrombus, the optimal preventive strategy remains uncertain. Some experts advocate observation alone in the absence of thrombus or neurological symptoms, whereas others suggest antiplatelet therapy due to the theoretical risk of thrombus formation within the flow-disturbance zone created by the web. In our patient, the presence of modifiable vascular risk factors and a strong family history of ischemic stroke influenced the decision to initiate antiplatelet therapy as a preventive measure. In symptomatic patients, multiple observational studies suggest that both carotid endarterectomy and stenting have excellent outcomes in preventing future events [11-12]. However, the absence of randomized controlled trials limits definitive conclusions.

The 2021 American Heart Association/American Stroke Association guideline for prevention of stroke in patients with stroke or transient ischemic attack recommends antiplatelet therapy (Class I, Level B-NR) in patients with carotid webs within the vascular territory of ischemic stroke or transient ischemic attack in whom no alternative cause of stroke is identified [13]. Furthermore, in cases of recurrent strokes while on medical treatment and no other cause of stroke can be identified, surgical options such as endarterectomy or stenting of the carotid artery can be proposed (Class IIb, Level C-LD) [13].

There are no specific recommendations in the 2023 European Society for Vascular Surgery guidelines for symptomatic individuals with carotid webs, as these guidelines focus solely on atherosclerosis in the carotid arteries. However, based on observational data, several invasive treatment options for carotid web have been described [4,10-12].

Neither the American Heart Association/American Stroke Association nor the European Society for Vascular Surgery provides recommendations regarding the treatment of asymptomatic carotid webs. Therefore, the management of these patients should be individualized. In asymptomatic patients without thrombus or significant stenosis, conservative management, including antiplatelet therapy and aggressive cardiovascular risk factor modification, is generally preferred. However, there is currently limited evidence available to support this approach, and most of it has been extrapolated from studies on stroke prevention.

In our patient, the carotid web was detected incidentally during vascular evaluation and was not associated with neurological symptoms or intraluminal thrombosis. Nevertheless, our patient had several modifiable vascular risk factors, particularly active smoking, and a notable family history of ischemic stroke. As a result, we decided to manage this patient conservatively with an antiplatelet agent and an aggressive lifestyle modification program. Smoking cessation and regular physical activity were strongly recommended to reduce overall cardiovascular risk.

Our case highlights several important clinical aspects. First, carotid webs should be recognized as significant non-atherosclerotic abnormalities of the carotid bulb that may be found incidentally during DUS. Second, awareness of their characteristic imaging features is essential to differentiate them from other carotid pathologies. Finally, this case illustrates the gap in knowledge surrounding the management of asymptomatic carotid webs, emphasizing the need for further prospective studies to better define pathogenesis and evidence-based treatment options.

This case report has several limitations arising mainly from the fact that it is a single case observation. In addition, the carotid web was discovered incidentally in an asymptomatic patient on DUS and CTA without histopathological confirmation, as invasive treatment was not indicated in this case.

## Conclusions

Carotid web is an uncommon non-atherosclerotic abnormality of the carotid artery that may increase the risk of embolic stroke even in the absence of significant luminal stenosis.

Current guidelines provide recommendations for symptomatic patients but not for incidental asymptomatic cases. Pending stronger evidence, individualized management with antiplatelet therapy and optimization of cardiovascular risk factors may represent a reasonable approach.
